# Case report: Genotype-phenotype characteristics of nine novel *PKD1* mutations in eight Chinese patients with autosomal dominant polycystic kidney disease

**DOI:** 10.3389/fmed.2023.1268307

**Published:** 2023-10-11

**Authors:** Jing Zhuang, Ailima Aierken, Dilina Yalikun, Jun Zhang, Xiaoqin Wang, Yongfang Ren, Xuefei Tian, Hong Jiang

**Affiliations:** ^1^Division of Nephrology, Department of Internal Medicine, People’s Hospital of Xinjiang Uygur Autonomous Region, Ürümqi, China; ^2^Department of Radiology and Medical Imaging, People’s Hospital of Xinjiang Uygur Autonomous Region, Ürümqi, China; ^3^Section of Nephrology, Department of Internal Medicine, Yale University School of Medicine, New Haven, CT, United States

**Keywords:** second-generation sequencing, autosomal dominant polycystic kidney disease, *PKD1* gene mutation, genotype-phenotype characteristics, genetic kidney disease

## Abstract

**Introduction:**

Autosomal dominant polycystic kidney disease (ADPKD) is a common genetic disorder. The *PKD1* gene is responsible for the majority of ADPKD cases, and the mutations in this gene exhibit high genetic diversity. This study aimed to investigate the association between genotype and phenotype in ADPKD patients with *PKD1* gene mutations through pedigree analysis.

**Methods:**

Eight Chinese pedigrees affected by ADPKD were analyzed using whole-exome sequencing (WES) on peripheral blood DNA. The identified variants were validated using Sanger sequencing, and clinical data from the patients and their families were collected and analyzed.

**Results:**

Nine novel mutation sites in *PKD1* were discovered across the pedigrees, including c.4247T > G, c.3298_3301delGAGT, c.4798A > G, c.7567G > A, c.11717G > C, c.7703 + 5G > C, c.3296G > A, c.8515_8516insG, and c.5524C > A. These mutations were found to be associated with a range of clinical phenotypes, including chronic kidney disease, hypertension, and polycystic liver. The age of onset and disease progression displayed significant heterogeneity among the pedigrees, with some individuals exhibiting early onset and rapid disease progression, while others remained asymptomatic or had milder disease symptoms. Inheritance patterns supported autosomal dominant inheritance, as affected individuals inherited the mutations from affected parents. However, there were instances of individuals carrying the mutations who remained asymptomatic or exhibited milder disease phenotypes.

**Conclusion:**

This study highlights the importance of comprehensive genotype analysis in understanding the progression and prognosis of ADPKD. The identification of novel mutation sites expands our knowledge of *PKD1* gene mutations. These findings contribute to a better understanding of the disease and may have implications for personalized therapeutic strategies.

## Introduction

Polycystic kidney disease (PKD) is a genetic disease that can be inherited in two different ways: autosomal dominant inheritance and autosomal recessive inheritance. Autosomal dominant polycystic kidney disease (ADPKD) is the most common single-gene hereditary kidney disease, with an incidence rate of about 1/1000-1/2500 (1, 2). ADPKD is classified into two types, polycystic kidney disease type 1 (OMIM173900) and polycystic kidney disease type 2 (OMIM613095). The discovery and cloning of the *PKD1* and *PKD2* genes have facilitated the development of molecular diagnostics based on DNA sequencing (3). About 78% of ADPKD cases are caused by *PKD1* gene mutations on chromosome 16, and about 15% of cases are caused by *PKD2* gene mutations on chromosome 4 (4, 5). The *PKD1* and *PKD2* genes encode polycystin 1 (PC1) and polycystin 2 (PC2), respectively, which are primarily located in the primary cilium of renal tubular epithelial cells. In addition to mutations in the *PKD1* and *PKD2* genes, other mutations have been found in some ADPKD patients, including *GANAB*, *DNAJB11*, *IFT140* and other gene mutations that can cause atypical ADPKD (6–8). Mutations in genes related to autosomal dominant polycystic liver disease (*PRKCSH*, *SEC63*, *LRP5*, *ALG8*, and *SEC61B*) may also cause mild kidney phenotypes (9). Clinical manifestations of ADPKD include hypertension, hematuria, proteinuria, and decreased kidney function. Patients with ADPKD may also experience other complications involving multiple organs or tissues, such as liver cysts, pancreatic cysts, spleen cysts, and intracranial aneurysms.

About 50% of ADPKD patients usually progress to end-stage kidney disease (ESKD) by the age of 60 (10, 11). Evidence shows that about 5−10% of patients receiving kidney replacement therapy (KRT) worldwide have ADPKD, and it accounts for about 5% of ESKD patients in China, affecting about 1.25 million people (12). ADPKD is the fourth most common cause of ESKD, and it brings a heavy burden to society and families (3).

Currently, there have been significant advancements in understanding the pathogenesis of ADPKD in the fields of cell biology and molecular mechanisms. Mutations in the main genes *PKD1* and *PKD2* lead to various abnormalities in downstream cell signal transduction, including intracellular calcium disorders, abnormalities in cAMP and mTOR signaling pathways, and classical and non-classical Wnt pathway signal activation. These abnormalities partially explain the pathogenesis and progression of kidney cysts. In addition to the genetically abnormal PKD genes, several studies have shown that the formation of cysts requires the normal haplotype of secondary acquired somatic cell loss, known as the “second hit” hypothesis. Additionally, a “threshold” model for cyst formation has been proposed, suggesting that cyst formation may be triggered when the level of polycystin protein in renal tubular epithelial cells falls below a critical threshold (13). Despite significant progress in understanding the pathogenesis of ADPKD, several issues remain unresolved. For instance, while PC2 is known to function as a TRP-like calcium channel and interact with PC1 to regulate primary cilia signaling pathways, the exact role of calcium ions in the pathogenesis of ADPKD is still not fully understood. Moreover, the mechanism by which decreased polycystin signaling in cilia leads to the formation of cystic lesions remains unclear, highlighting the need for further basic and clinical research to expand our understanding of ADPKD’s pathogenesis (13, 14).

Early diagnosis of PKD patients, identification of pathogenic mutations in ADPKD families, regular monitoring of clinical indicators, and timely symptomatic treatment can help delay the progression of ADPKD. Genetic diagnosis is increasingly being used to provide information for the clinical management of PKD nephropathy patients. This approach can result in a more accurate diagnosis, targeted disease monitoring, treatment, and family genetic counseling (15). Studies have shown a high correlation between the phenotype and genotype of ADPKD patients, with pathogenic gene mutations being the primary determinant of disease progression (16). Several studies have shown that the type of mutation is closely related to the severity of ADPKD. Patients with *PKD2* mutations generally have a milder form of kidney disease compared to those with *PKD1* mutations (17). Additionally, frameshift mutations, nonsense mutations, and splice site changes in *PKD1* truncated mutations are associated with more severe disease than non-truncated mutations, such as missense mutations and in-frame insertion/deletion (18–20). Furthermore, mutations in non-*PKD1* or *PKD2* genes can also result in the development of renal cysts. For example, the deletion of the tuberous sclerosis complex 2 (*TSC2*) gene adjacent to the *PKD1* gene can cause a rare and severe form of polycystic kidney disease that often leads to ESKD in childhood (21).

In some cases of ADPKD, mutations in *PKD1* or *PKD2* genes are not detected, and somatic mosaicism may be present (22). These mutations are not concentrated in any specific location of the gene. Identifying gene mutations associated with ADPKD can aid in diagnosis and predicting disease progression. Ultrasound, the most commonly used diagnostic method, may have low sensitivity, particularly in high-risk individuals under 40 years of age, hindering early or presymptomatic diagnosis (23). With the advancements in precision medicine and increased understanding of cystic diseases, whole-exome sequencing (WES) using next-generation sequencing may become a vital tool in screening for ADPKD and providing accurate diagnoses and effective treatments.

This study involved the use of whole-exome sequencing (WES) to screen eight Chinese patients who had been diagnosed with ADPKD and then verified the results with Sanger sequencing in their family members. We also analyzed the pathogenicity of the mutations to identify the specific pathogenic mutation gene responsible for the disease in each patient and their family. Some of the gene mutations identified in this study have not been previously reported, which has important implications for both clinical practice and further research on ADPKD.

## Materials and methods

### Patients

From November 2021 to November 2022, we conducted a study on ADPKD patients admitted to the People’s Hospital of Xinjiang Uygur Autonomous Region (Ürümqi, China). The diagnosis of ADPKD was made based on the criteria outlined in Table 1, as previously described (12). A total of eight patients from distinct families were enrolled in the study. Blood samples were obtained from each patient and their immediate family members via peripheral venous collection.

**TABLE 1:** Criteria for diagnosis and exclusion of autosomal dominant polycystic kidney disease.

Age, years	15−39	40−59	>60
Diagnostic confirmation	At least 3 cysts (unilateral or bilateral)	At least 2 cysts in each kidney	At least 4 cysts in each kidney
Disease exclusion	No recommendation	<2 cysts in each kidney	<2 cysts in each kidney

The study was approved by the Medical Ethics Committee of the People’s Hospital of the Autonomous Region and was conducted in compliance with the Declaration of Helsinki. The study involving human participants was reviewed and approved by the Medical Ethics Committee of the People’s Hospital of Xinjiang Uygur Autonomous Region, and written informed consent was obtained from all participants (Approval number: KY2023032201).

### Acquisition of clinical data

Abdominal ultrasonography, computed tomography (CT), or magnetic resonance imaging (MRI) were conducted on the proband and their family members to gather clinical data based on their medical history and clinical manifestations. Laboratory data, including blood and urine sample analysis, as well as biochemical tests, were also collected.

### Target gene enrichment, library construction, and capture sequencing

A peripheral blood sample (5 mL) was drawn from participants, and genomic DNA (gDNA) was extracted using the MagPure Buffy Coat DNA Midi KF Kit as per the manufacturer’s instructions. BGI’s enzyme kit (Segmentase, BGI) was used to fragment the gDNA into fragments of 100−500 bp, which were then subjected to magnetic bead selection to collect fragments of 280−320 bp. After repairing the ends of the fragments, “A” bases were added to the 3′ overhangs, enabling them to pair with a special adapter containing “T” bases. The library with adapters was then amplified using pre-capture ligation-mediated PCR (LM-PCR), purified, and enriched by array hybridization (Roche KAPA HyperExome, Madison, WI, USA) for 16−24 h at 47°C, followed by elution and post-capture amplification. The enriched products were assessed for the level of enrichment using the Agilent 2100 Bioanalyzer and BMG. The qualified products were pooled and quantified based on different library quantities, and the single-stranded library products were circularized to generate DNA nanoballs (DNBs) before being sequenced using PE100 + 100 sequencing on the MGISEQ-2000 platform.

### Analysis of sequencing results

Upon receipt of the primary sequencing data, we conducted bioinformatics processing and data analysis to identify potential variants in the family. To perform further analysis, we used previously established filtering criteria to generate “clean reads.” The “clean reads” were then aligned to the human genome reference (hg19 version) using the BWA Multi-Vision software package (0.7.17) and samtools (1.10). The output files from the alignment were used to conduct sequencing coverage and depth analysis of the target region, single-nucleotide variants (SNVs), and INDEL calling. To detect SNVs and indels, we utilized the GATK software (4.1.9.0), and all SNVs and indels were filtered and evaluated using multiple databases, including NCBI dbSNP, 1000 human genome dataset (phase 3), ClinVar (2020-03-16), ESP6500 (V2), GnomAD (v2.1.1), ExAC (r0.3.1), and SecondaryFinding_Var* (V1.1_2020.3, an in-house database of BGI Genomics). To predict the impact of missense variants, we employed dbNSFP, which consists of seven well-established *in silico* prediction programs such as SIFT, Polyphen2, LRT, MutationTaster, and PhyloP. To analyze the variants near the splicing sites, we used SpliceAI (1.3) for prediction. Lastly, variant classification was conducted following the American Society for Medical Genetics and Genomics (ACMG) guidelines, and reported as pathogenic, likely pathogenic, variant of unknown significance (VUS), likely benign, or benign.

### Sanger verification

Conventional Sanger sequencing methods were used to validate all mutations and potential pathogenic variants. If DNA was available from family members, segregation analysis was performed (24).

## Results

### Clinical phenotype of the probands and their family members

Pedigree A includes a 48-year-old male proband who showed signs of chronic kidney disease stage 2 (CKD2) and hypertension (HT), as determined by clinical data. Abdominal CT results revealed an increase in the size of both kidneys with low and slightly high-density shadows of varying sizes. Multiple round low-density shadows in the liver suggested the presence of polycystic liver (PL) (Table 2). The proband’s father had also been diagnosed with ADPKD in middle age, and the proband’s son was found to be a carrier of the *PKD1* mutation gene but appeared to be asymptomatic, suggesting autosomal dominant inheritance in the family (Table 3).

**TABLE 2:** Clinical features of probands with autosomal dominant polycystic kidney disease.

Characteristics	Proband A	Proband B	Proband C	Proband D	Proband E	Proband F	Proband G	Proband H
Sex	Male	Female	Female	Female	Male	Male	Female	Male
Age (years)	48	50	55	33	49	44	58	32
Age at diagnosis (years)	36	43	32	31	38	36	28	17
Age at ESKD (years)	N	N	45	N	38	43	58	N
Family history	Y	Y	Y	Y	Y	Y	Y	Y
Stage of CKD	CKD2	CKD2	CKD5	CKD3	CKD5	CKD5	CKD5	CKD2
HT (age at diagnosis)	38	43	44	N	29	36	28	32
Complication	PL	PL IAC	PL	OC	PL EC	PL	PL	PL
BMI (kg/m^2^)	24.22	27.83	23.44	20.05	28.95	22.84	26.91	25.38
Scr (μmol/L)	105.30	74.00	754.90	111.90	929.80	582.40	570.50	103.40
BUN (mmol/L)	7.13	6.24	16.00	6.07	20.10	18.33	16.22	7.27
UA (μmol/L)	433.00	295.00	394.05	390.00	465.00	248.86	346.38	358.64
eGFR (ml/min × 1.73 m^2^)	60.30	74.00	6.31	50.69	7.74	11.28	8.57	76.41
Hematuria	N	2+	2+	N	1+	N	1+	2+
Microalbuminuria (mg/L)	15.30	16.70	–	206.70	–	176.30	113.00	76.70

CKD, chronic kidney disease; HT, hypertension; ESKD, end-stage kidney disease; BMI, body mass index. Scr, serum creatinine, BUN, blood urea nitrogen; UA, uric acid; eGFR, estimated glomerular filtration rate calculated by MDRD formula; PL, polycystic liver disease; IAC, intracranial arachnoid cyst; OC, ovarian cyst; EC, epididymal cyst.

**TABLE 3:** Family history in probands with autosomal dominant polycystic kidney disease.

Proband	Clinical characteristics at the time of diagnosis		Family history			
	Age (years)	Clinical manifestation	Father	Mother	Sibling(s)	Children
Proband A	36	Routine physical examination (US)	ADPKD	no ADPKD	No ADPKD	Son is asymptomatic, but has *PKD1* mutation
Proband B	43	Pain (US)	Deceased	ADPKD, deceased	An elder sister with ADPKD, PL	No child
Proband C	32	Routine physical examination (US)	Deceased	ADPKD, deceased	A younger brother and a younger sister with ADPKD	A son with ADPKD
Proband D	31	Pregnancy (US)	ADPKD	No ADPKD	No ADPKD	Son is asymptomatic, but has *PKD1* mutation
Proband E	38	Routine physical examination (US)	Deceased	ADPKD, deceased	An elder sister with ADPKD	no ADPKD
Proband F	43	Routine physical examination (US)	ADPKD, deceased	No ADPKD	A younger brother and a younger sister with ADPKD	no ADPKD
Proband G	28	Routine physical examination (US)	Deceased	Deceased	No ADPKD	A daughter with ADPKD
Proband H	17	Routine physical examination (US)	ADPKD, deceased	No ADPKD	No ADPKD	A son with several cysts in kidneys

US, ultrasound; PL, polycystic liver disease.

Pedigree B includes a 50-year-old female proband who showed symptoms of CKD2 with hematuria, HT, PL, and intracranial arachnoid cyst (IAC). Imaging results revealed an increase in the size of both kidneys with many anechoic areas of varying sizes, as well as an abnormal liver with anechoic areas of different sizes. Cranial MRI showed arachnoid cysts in the right forehead and left temporal region (Table 2). The proband’s mother and sister were also diagnosed with ADPKD, and the nephew was found to carry the *PKD1* mutation gene but was asymptomatic, indicating autosomal dominant inheritance in the family (Table 3).

Pedigree C includes a 55-year-old female proband who showed signs of CKD5 with hematuria, HT, and PL. Abdominal CT scan showed enlarged bilateral kidneys with kidney cysts, it also showed innumerable cysts in the liver (Table 2). In this family, the proband’s mother, younger brother, younger sister, and son were diagnosed with ADPKD, and the proband’s mother had passed away (Table 3). These findings suggest an autosomal dominant inheritance pattern in the family.

In pedigree D, a 33-year-old female proband displayed CKD3 symptoms and ovarian cyst (OC). Abdominal ultrasound results showed normal size and shape of both kidneys, but there were many anechoic areas of different sizes (Table 2). The proband’s father was diagnosed with ADPKD, and the son was a carrier of the *PKD1* mutation gene but appeared normal, indicating that the inheritance pattern is autosomal dominant in the family (Table 3).

In pedigree E, a 49-year-old male proband presented with CKD5 with hematuria, HT, PL, and epididymal cyst (EC). Abdominal ultrasound showed that the right kidney was significantly enlarged with multiple anechoic areas of varying sizes. The left kidney had been removed due to a renal cyst rupture and hemorrhage 4°years prior (Table 2). The proband’s mother and elder sister both have ADPKD, and the proband’s mother passed away. However, the proband’s child did not inherit the disease. These findings suggest an autosomal dominant inheritance pattern in the family (Table 3).

In pedigree F, a 44-year-old male proband was diagnosed with CKD5, PL, and HT. Abdominal ultrasound showed significantly enlarged kidneys with multiple anechoic areas of different sizes and anechoic lesions in the right lobe of the liver (Table 2). The proband’s father had ADPKD and passed away, and the proband’s younger brother and younger sister were also diagnosed with ADPKD (Table 3).

In pedigree G, a 58-year-old female proband presented with CKD5 with hematuria, HT, and PL. Abdominal ultrasound showed increased kidney size with multiple anechoic areas, as well as several anechoic lesions in the liver (Table 2). The proband’s daughter was diagnosed with ADPKD, while her siblings did not have the condition (Table 3).

In pedigree H, a 32-year-old male proband presented with CKD2 with hematuria, HT, and PL. Abdominal ultrasound showed enlarged kidneys with multiple anechoic areas and anechoic lesions in the liver (Table 2). The proband’s father had a history of ADPKD and passed away, and his son was a carrier of the *PKD1* mutation gene with several kidney cysts despite having a normal appearance, suggesting an autosomal dominant inheritance pattern (Table 3).

### Genotype of the probands and their family members

**FIGURE 1:**
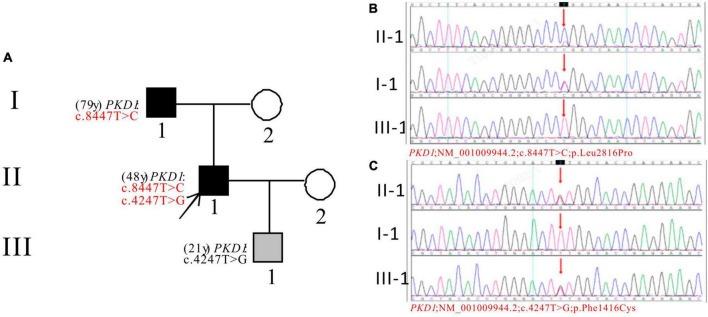
Pedigree diagram and sequencing results of proband A. **(A)** Pedigree diagram. The WES identified a heterozygous missense mutation in exon 23 (c.8447T > C) **(B)** and a heterozygous missense mutation in exon 15 (c.4247T > G) **(C)** of the *PKD1* gene in the proband (II-1) and affected family members (I-1 and III-1). The mutation sites were indicated by a red arrows in panels **(B,C)**.

In Pedigree A, WES analysis revealed that the proband had one likely pathogenic mutation and one uncertain mutation in the *PKD1* gene (Supplementary Table 1). The analysis showed a heterozygous missense mutation in exon 23 of *PKD1*: c.8447T > C (p.Leu2816Pro), which has been reported as likely pathogenic (25–27) and is also indicated by the ACMG guidelines. Another heterozygous missense mutation was detected in exon 15 of *PKD1*: c.4247T > G (p.Phe1416Cys). There is no relevant literature on the pathogenicity of this variation. Sanger sequencing confirmed that proband’s father (I-1) was a PKD patient (79 years old) with *PKD1*: c.8447T > C, while III-1 (21 years old) carries *PKD1*: c.4247T > G and is currently an asymptomatic carrier of the mutation (Figure 1).

**FIGURE 2:**
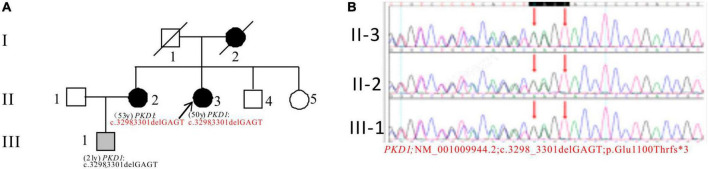
Pedigree diagram and sequencing results of proband B. **(A)** Pedigree diagram. **(B)** WES identified a heterozygous deletion mutation in exon 15 (c.3298_3301delGAGT) of the *PKD1* gene in the proband (II-3) and affected family members (II-2 and III-1). The mutation sites were indicated by a red arrows panel in panel **(B)**.

In Pedigree B, WES analysis revealed a likely pathogenic mutation in the *PKD1* gene in the proband (Supplementary Table 1). Specifically, a heterozygous deletion mutation in exon 15 of *PKD1* was identified: c.3298_3301delGAGT (p.Glu1100Thrfs*3). Sanger sequencing was performed to verify the results and it was found that the proband’s sister (II-2) (53 years old) also carries the same mutation and was diagnosed with ADPKD at the age of 45. Her kidney function began to decline at the age of 52, reaching CKD3. Additionally, the proband’s nephew (III-1) (21 years old) seemingly asymptomatic, but has the same *PKD1* mutation (Figure 2).

**FIGURE 3:**
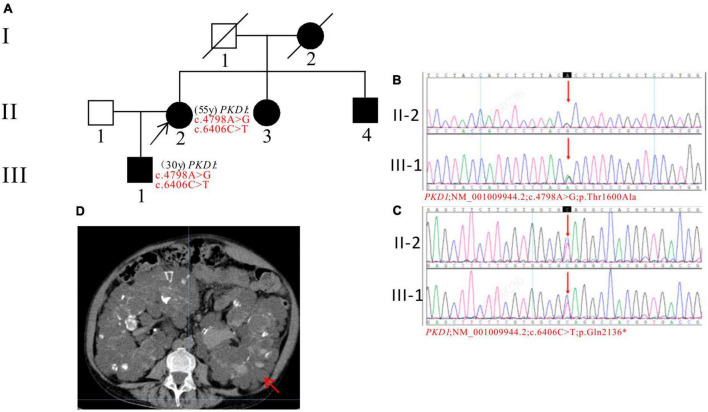
Pedigree diagram, sequencing and abdominal CT scan result of proband C. **(A)** Pedigree diagram. The WES identified a heterozygous nonsense mutation in exon 15 (c.6406C > T) **(B)** and a heterozygous missense mutation (c.4798A > G) **(C)** of the *PKD1* gene in the proband (II-2) and affected family member (III-1). The mutation sites were indicated by a red arrows in panels **(B,C)**. **(D)** Abdominal CT scan performed in proband C at 55y showed enlarged bilateral kidneys with kidney cysts. The kidney cysts is indicated by a red arrow.

In Pedigree C, WES analysis revealed a pathogenic mutation and an uncertain mutation in the *PKD1* gene in the proband (Supplementary Table 1). Specifically, a heterozygous nonsense mutation in exon 15 of *PKD1* was identified: c.6406C > T (p.Gln2136*), which has been reported as pathogenic (28) and is also indicated by the ACMG guidelines. Another heterozygous missense mutation was also detected in exon 15 of *PKD1*: c.4798A > G (p.Thr1600Ala), which has an uncertain significance according to the ACMG guidelines and currently lacks reports on its pathogenicity. Sanger sequencing confirmed that the proband’s son (III-1) (30 years old) carried both mutations and was diagnosed with ADPKD at the age of 20. His kidney function began to decline at the age of 29, reaching CKD2. The proband’s mother (I-2), sister (II-3), and brother (II-4) were all diagnosed with ADPKD. Among them, the proband’s sister (II-3) reached ESKD at the age of 45, received a kidney transplant at the age of 52, and is currently in generally good condition (Figure 3).

**FIGURE 4:**
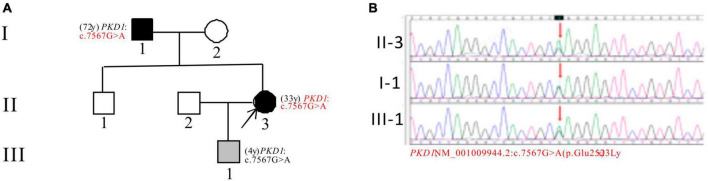
Pedigree diagram and sequencing results of proband D. **(A)** Pedigree diagram. **(B)** WES identified a heterozygous missense mutation in exon 19 (c.7567G > A) of the *PKD1* gene in the proband (II-3) and affected family members (I-1 and III-1). The mutation sites were indicated by a red arrows in panel **(B)**.

In Pedigree D, WES analysis revealed a mutation in the *PKD1* gene in the proband (Supplementary Table 1). Specifically, a heterozygous missense mutation in exon 19 of the *PKD1* gene was identified: c.7567G > A (p.Glu2523Lys), which is of unknown significance according to the ACMG guidelines and currently lacks reports on its pathogenicity. Sanger sequencing confirmed that the father of the proband (I-1) (72 years old) and the proband’s son (III-1) (4 years old) carried the same mutation. I-1 was diagnosed with PKD at the age of 72 and currently has normal kidney function, while III-1 is seemingly asymptomatic now (Figure 4).

**FIGURE 5:**
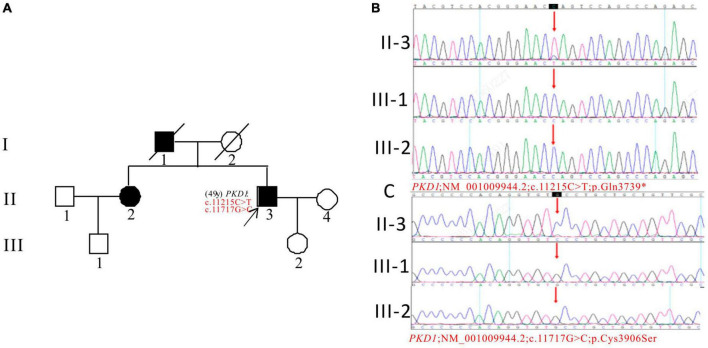
Pedigree diagram and sequencing results of proband E. **(A)** Pedigree diagram. The WES identified a heterozygous nonsense mutation in exon 39 (c.11215C > T) **(B)** and a heterozygous missense mutation (c.11717G > C) in exon 43 **(C)** of the *PKD1* gene in the proband, and the results of family members (III-1 and III-2) were displayed. The mutation sites were indicated by a red arrows in panels **(B,C)**.

In Pedigree E, the WES analysis of the proband revealed a pathogenic variation and a uncertain variation in the *PKD1* gene (Supplementary Table 1). Specifically, a heterozygous nonsense mutation in exon 39 of *PKD1* was identified: c.11215C > T (p.Gln3739*), which has been classified as pathogenic according to the ACMG guidelines. Another heterozygous missense mutation was detected in exon 43 of *PKD1*: c.11717G > C (p.Cys3906Ser), which has an uncertain significance according to the ACMG guidelines and currently lacks reports on its pathogenicity. The proband’s father (I-1) and sister (II-2) were diagnosed with ADPKD. However, the corresponding *PKD1* gene mutation was negative for the proband’s son (III-2) (11 years old) and proband’s nephew (III-1) (29 years old), both of whom were apparently normal population (Figure 5).

**FIGURE 6:**
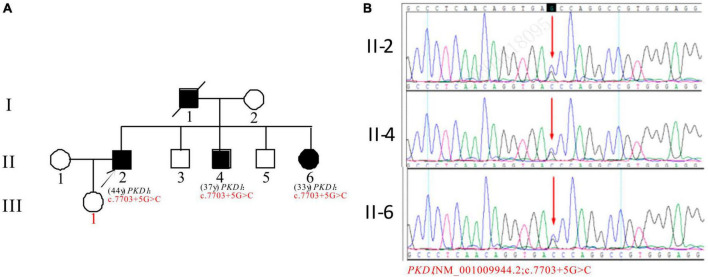
Pedigree diagram and sequencing results of proband F. **(A)** Pedigree diagram. **(B)** A heterozygous intron variation (c.7703 + 5G > C) of the *PKD1* gene in the proband (II-2) and affected family members (II-4 and II-6) were identified. The mutation sites are indicated by a red arrows in panel **(B)**.

In Pedigree F, the proband was found to have a heterozygous intronic mutation in the *PKD1* gene: c.7703 + 5G > C (Supplementary Table 1). This mutation is located within an intron of the gene and its significance is currently unknown according to the ACMG guidelines. There are no reports available regarding its pathogenicity. The proband’s father (I-1) was a PKD patient who passed away. Sanger sequencing confirmed that one of the proband’s brothers (II-4) (37 years old) and sisters (II-6) (33 years old) carried the same mutation. II-4 was diagnosed with ADPKD at 36 years old, entered ESKD at the age of 37. II-6 was diagnosed with ADPKD at the age of 32 and currently maintains normal kidney function. However, the mutation in the *PKD1* gene was not detected in the proband’s daughter (III-1) (16 years old) (Figure 6).

**FIGURE 7:**
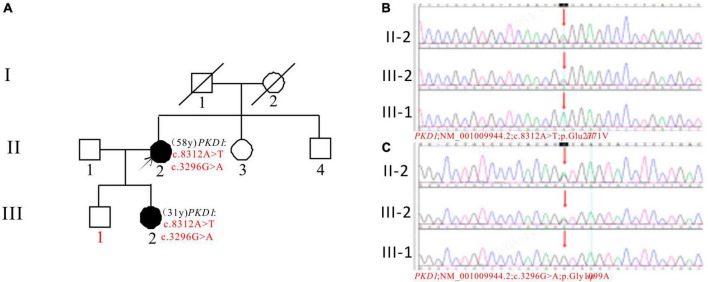
Pedigree diagram and sequencing results of proband G. **(A)** Pedigree diagram. The WES identified a heterozygous missense mutation in exon 23 (c.8312A > T) **(B)** and a heterozygous missense mutation in exon 15 (c.3296G > A) **(C)** of the *PKD1* gene in the proband (II-2), and the results in family members (III-1 and III-2) were displayed. The mutation sites are indicated by a red arrows in panels **(B,C)**.

In Pedigree G, WES analysis revealed a likely pathogenic mutation and an uncertain mutation in the *PKD1* gene (Supplementary Table 1). Specifically, a heterozygous missense mutation in exon 23 of the *PKD1* gene was identified: c.8312A > T (p.Glu2771Val), which has been indicated as likely pathogenic by the ACMG guidelines, and its pathogenicity had been reported (29). Another heterozygous missense mutation was detected in exon 15 of *PKD1*: c.3296G > A (p.Gly1099Asp), which is of unknown significance according to the ACMG guidelines and currently lacks reports on its pathogenicity. Sanger sequencing confirmed that the proband’s daughter (III-2) (31 years old) was a ADPKD patient with the same mutations and is currently with normal kidney function. The corresponding *PKD* gene mutation in the son of the proband (III-1) (36 years old) was negative (Figure 7).

**FIGURE 8:**
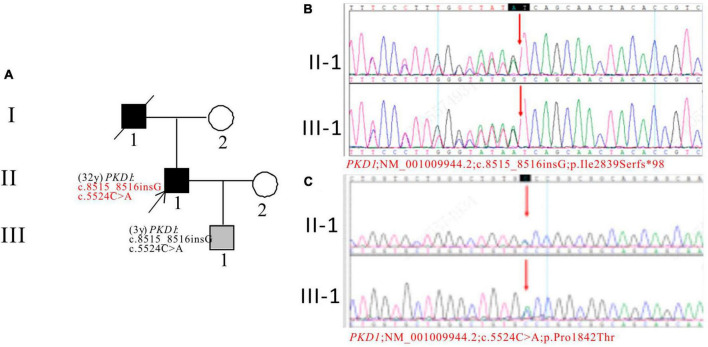
Pedigree diagram and sequencing results of proband H. **(A)** Pedigree diagram. The WES identified a heterozygous frameshift mutation in exon 23 (c.8515_8516insG) **(B)** and a heterozygous missense mutation in exon 15 (c.5524C > A) **(C)** of the *PKD1* gene in the proband (II-1) and affected family member (III-1). The mutation sites were indicated by a red arrows in panels **(B,C)**.

In Pedigree H, WES analysis revealed a pathogenic and an uncertain mutation in the *PKD1* gene (Supplementary Table 1). Specifically, a heterozygous frameshift mutation in exon 23 of the *PKD1* gene was identified: c.8515_8516insG (p.Ile2839Serfs*98), which has been indicated as pathogenic by the ACMG guidelines. Another heterozygous missense mutation was detected in exon 15 of *PKD1*: c.5524C > A (p.Pro1842Thr), which has been indicated as having uncertain significance according to the ACMG guidelines. At present, there are no reports on their pathogenicities. The proband’s father was an ADPKD patient who passed away at the age of 50. Sanger sequencing confirmed that the son of the proband (III-1) (3 years old) carries the mutations and is an apparently normal mutation carrier (Figure 8).

## Discussion

In this study, we aimed to analyze the gene mutations in 8 Chinese families with ADPKD. This investigation is crucial to expand the genetic diversity data of different ethnic groups, improve the ADPKD mutation database, and explore its underlying pathogenesis. Due to the differences in genetic backgrounds and environmental factors, there is considerable phenotypic variability among ADPKD patients. Key factors that determine the progression of ADPKD include age, PKD genotype (including mutation gene, mutation type, gene mutation location, and the presence of multiple gene mutations), urine protein, estimated glomerular filtration rate (eGFR), and highly adjusted total kidney volume (HtTKV) (30).

Two variants of the *PKD1* gene were found in Pedigree A: c.8447T > C (p.Leu2816Pro) and c.4247T > G (p.Phe1416Cys). The first variant is known to cause ADPKD in several patients and families (26, 27). The pathogenicity of the second variant is unknown, and no information on its frequency in the population was found in the GnomAD databases. The proband and their father had ADPKD, with the father having the pathogenic mutation. The proband’s son is a carrier of the second variant with a normal phenotype. Further study is needed to determine the role of the second variant in ADPKD.

Pedigree B’s proband was diagnosed with ADPKD and has a frameshift mutation c.3298_3301delGAGT (p.Glu1100Thrfs*3) in *PKD1* gene. The mutation is not reported in relevant databases, and leads to a truncated PC1 protein affecting its function (31). No information on its frequency in the population was found in the GnomAD databases. The mutation is suspected to be pathogenic based on ACMG guidelines and is inherited from the proband’s mother who died of ADPKD. The proband’s sister also has ADPKD and PL. The proband’s nephew carries the mutation but is currently asymptomatic. Further follow-up is ongoing.

In pedigree C, two variants were found in the *PKD1* gene. One of the variants, c.6406C > T, has been reported as pathogenic (28). The other variant, c.4798A > G, is new and its pathogenicity is uncertain. The population frequency in the GnomAD is 0.000016. The variants may work together to cause the disease in the family. Family members with the variants show co-segregation of genotype and phenotype, with early onset of ADPKD and kidney failure. The role of the new variant in ADPKD needs further confirmation.

A new *PKD1* gene mutation c.7567G > A (p.Glu2523Lys) was detected in pedigree D, with the proband, father, and son carrying it. The proband has ADPKD and CKD3, while the father was diagnosed with ADPKD in middle age and has normal kidney function. The mutation is predicted to have harmful effects on the gene and is considered pathogenic in this family, although its significance is uncertain according to ACMG guidelines. The population frequency of this mutation in the GnomAD is 0.000051. The proband’s son is a carrier and is being closely monitored. Further research is needed to determine the mutation’s role in ADPKD.

The proband in pedigree E have *PKD1* gene mutations, including a harmful c.11215C > T(p.Gln3739*) nonsense mutation in exon 39 and a newly discovered c.11717G > C(p.Cys3906Ser) missense mutation in exon 43, which is of uncertain significance. The mutation frequency of c.11717G > C in the GnomAD databases is 0.000011. The proband’s sister is an ADPKD patient. Their father had ADPKD, suggesting inheritance. The proband’s daughter and nephew did not inherit the mutations. Both proband and his sister developed ESKD at an early age, and the two mutations are considered pathogenic when present together.

In pedigree F, the *PKD1* gene of the proband and his younger brother with the disease was found to have a novel intron variation c.7703 + 5G > C. However, the proband’s daughter did not inherit these mutations. No reports on the mutation frequency of the population at this locus in the GnomAD databases. The mutation frequency of the population at this locus is unknown. Predictive analysis indicates harmful effects on the gene and mRNA splicing. The mutation is predicted to be pathogenic based on genetic characteristics and clinical manifestations. However, it is still uncertain based on ACMG guidelines. The proband and his siblings may have inherited the mutation from their father who had PKD. Further observation and follow-up are needed.

Pedigree G has a proband who developed ADPKD in their youth and entered ESKD 30 years later. The proband’s daughter has ADPKD but normal kidney function. One of the mutations, c.8312A > T (p.Glu2771Val), has been reported previously (29), while the other mutation, c.3296G > A, is likely a new mutation, the mutation frequency in the GnomAD databases is 0.000061. Both mutations are predicted to be harmful and affect *PKD1* mRNA splicing. According to ACMG guidelines, the mutations are classified as of uncertain significance. The proband’s son is negative for the mutations and apparently normal. The mutations are considered pathogenic and require further follow-up observation.

In pedigree H, the proband and his son have two mutations in *PKD1*: a frameshift mutation and a missense mutation, which are likely new mutations. The frameshift mutation is predicted to be harmful and classified as pathogenic, no reports on the mutation frequency of the population at this locus in the GnomAD databases. The missense mutation is predicted to be harmful by one software tool (SpliceAI) and polymorphic/neutral by others, with uncertain significance, the mutation frequency in the GnomAD databases is 0.000006 in the GnomAD databases. The proband’s father had ADPKD, suggesting inheritance of the mutations. The proband has early onset ADPKD and his son has a single renal cyst with normal liver and kidney functions, currently under monitoring.

**FIGURE 9:**
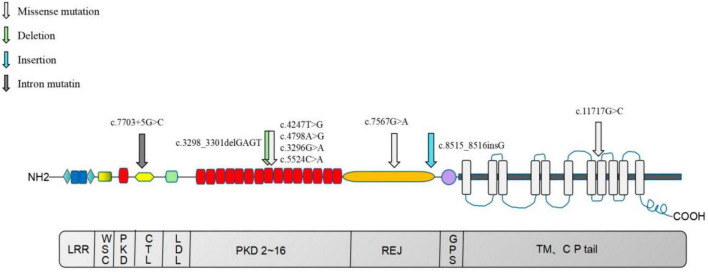
Distribution map of nine newly identified *PKD1* mutations in eight ADPKD patients. The upper section of the image illustrates the PC-1 domain, which includes 11 transmembrane domains, a large extracellular N-terminal domain, and a short intracellular C-terminal tail region. The lower section of the image depicts the coding region of the *PKD1* gene. LRR, Leucine-rich repeats; PKD, PKD functional area; CTL, C-type lectin motif, C-type phytohemagglutinin region; LDL, LDL-A like domain, rich in cysteine low-density lipoprotein receptor region; REJ, receptor egg jelly, the homologous region of sea urchin egg glue receptor; GPS, G-proteolytic site; TM, transmembrane, transmembrane domain; CP tail, cytoplasmic tail.

In this study, 13 mutation sites were identified in the *PKD1* gene, including 9 new mutations not previously reported, from 8 ADPKD probands (Figure 9). Among the 8 probands, 4 had progressed to ESKD, with ages ranging from 38 to 58 years old, and all had chosen hemodialysis as their kidney replacement therapy. Hypertension is a common occurrence in ADPKD patients, with about 60% diagnosed before kidney function decline (32, 33). The average age of hypertension diagnosis in *PKD1* mutation patients is around 39 years old (26). Seven of our 8 probands had hypertension, except for probands E and D, who were diagnosed at the same time or after the ADPKD diagnosis. It remains uncertain whether hypertension in ADPKD is caused by the primary angiopathy of polycystic protein gene mutation or the activation of a renal renin-angiotensin-aldosterone system (RAAS) due to cyst expansion, and further research is needed. Therefore, gene detection can offer valuable insights for the diagnosis and treatment of ADPKD with hypertension. ADPKD typically occurs after the age of 40 and 100% by age 80, making it a late-onset disease (34). In this study, *PKD1*-related mutations were also found in family members who appeared clinically normal and were younger than 21 years old. The absence of related clinical manifestations in these family members can be explained by the age-dependent delayed dominance of ADPKD, which requires further follow-up and observation.

The human *PKD1* gene is made up of 46 exons with complex structures. However, the presence of six pseudogenes, which are highly homologous to exons 1 to 33 of *PKD1*, poses a challenge to the molecular diagnosis of ADPKD (35). Second-generation sequencing technology has revolutionized genetic analysis, enabling disease group screening, WES, and even whole genome analysis, replacing single gene analysis based on Sanger sequencing. To date, the ADPKD mutation database has recorded 2,322 pathogenic *PKD1* mutations. However, most of these mutations occur only once in a single family, with few duplications. Therefore, it is necessary to investigate more families to confirm known mutations and discover new ones. Gene decoding-based basic and clinical studies of related drugs have shown that cystic disease progression can be delayed to varying degrees. Identifying specific pathogenic genes and alleles can help determine the disease prognosis, influencing disease management and the formulation of treatment plans (9, 36). If gene testing can help predict the benefits that drug therapy, represented by tolvaptan, can bring to ADPKD, it can create greater value for patients (37). However, slowing down cyst progression is not a long-term solution. The fundamental strategy is to reverse the functional re-expression of the pathogenic PKD gene at the physiological level *in vivo*. Stefan Somlo’s research team at Yale University has established a mouse model of PKD inactivation and reactivation to examine the impact of PKD inactivation on renal structure and function. They found that the re-expression of the PKD gene in the cystic kidney can rapidly reverse ADPKD, reducing cyst cell proliferation, activating autophagy, and restoring the cystic tubules to a normal lumen lined by cubic cells. Since the ADPKD phenotype is reversible, at least partially controlled by the function of the ADPKD gene (38), gene screening plays a crucial role in early diagnosis and intervention. Early diagnosis can benefit patients through lifestyle changes, drug intervention, and understanding of kidney transplantation information.

Kidney transplantation is considered the most effective surgical treatment for end-stage ADPKD (39, 40). Studies have shown that the recurrence rate of ADPKD after transplantation is similar to that of other non-diabetic kidney transplant recipients (41). Moreover, the volume of an autologous polycystic kidney tends to decrease after kidney transplantation (42). Therefore, in cases where there is enough space for kidney transplantation and no risk of bleeding or infection, it may not be necessary to remove the other kidney, which can improve the quality of life of patients. In ADPKD families, gene testing can help select suitable unaffected relatives as potential kidney transplant donors, promoting more transplantation evaluations and preemptive transplantation opportunities for ADPKD patients (43). This approach can improve patient outcomes, as preemptive transplantation before the onset of end-stage renal disease can prevent the need for dialysis, reduce post-operative complications, and improve long-term patient survival rates.

In this study, DNA was extracted from the subject’s blood, but the possibility of interpretation bias caused by chimerism cannot be ruled out. Moreover, the sample size was too small to eliminate all possible biases. In addition, this study did not provide a detailed molecular-level understanding of how new genes affect the functional changes of PC1. Therefore, larger clinical and basic research studies are needed to support more comprehensive genetic testing of ADPKD patients. Follow-up studies of patients and their family members have been extended to observe the relationship between genotype and phenotype more closely over time.

In summary, the findings from this study contribute to the growing body of knowledge on ADPKD genetics and highlight the importance of genetic testing in the diagnosis and management of this disease. By identifying new mutation sites and analyzing their effects on genotype and phenotype, this research provides a better understanding of ADPKD pathogenesis and opens up new avenues for further exploration. Second-generation sequencing technology has proven to be a valuable tool for detecting gene mutations in ADPKD patients, and its use should be encouraged in clinical practice and research. Ultimately, genetic testing can lead to more accurate diagnoses, better disease management, and improved genetic counseling for affected families. Further studies are needed to confirm and expand upon these findings and to elucidate the underlying mechanisms of ADPKD pathogenesis.

## Data availability statement

The datasets presented in this study can be found in online repositories. The names of the repository/repositories and accession number(s) can be found below: The National Omics Data Encyclopedia (NODE), accession number: OEP004096.

## Ethics statement

The studies involving humans were approved by the Ethics Committee of the Xinjiang Uygur Autonomous Region People’s Hospital confirms the approval of the project titled “Genotype-Phenotype Characteristics of Nine Novel PKD1 Mutations in Eight Chinese Patients with Autosomal Dominant Polycystic Kidney Disease” with the approval number KY2023032201. The studies were conducted in accordance with the local legislation and institutional requirements. Written informed consent for participation in this study was provided by the participants’ legal guardians/next of kin. Written informed consent was obtained from the individual(s), and minor(s)’ legal guardian/next of kin, for the publication of any potentially identifiable images or data included in this article.

## Author contributions

HJ: Conceptualization, Investigation, Methodology, Validation, Supervision, Data curation, Resources, Writing – review and editing, Funding acquisition. XT: Conceptualization, Methodology, Validation, Supervision, Writing – review and editing. JiZ: Investigation, Data curation, Writing – original draft, Methodology, Formal analysis, Software, Resources, Validation. AA: Investigation, Data curation, Writing – original draft, Methodology, Formal analysis, Software, Resources, Validation. DY: Investigation, Methodology, Data curation, Formal analysis, Writing – review and editing. JuZ: Investigation, Methodology, Data curation, Formal analysis, Writing – review and editing. XW: Investigation, Methodology, Data curation, Formal analysis, Writing – review and editing. YR: Investigation, Visualization, Writing – review and editing.
