# Supplementary material for: Case report: Genotype-phenotype characteristics of nine novel PKD1 mutations in eight Chinese patients with autosomal dominant polycystic kidney disease
Source: Front Med (Lausanne). 2023 Oct 11;10:1268307. doi: 10.3389/fmed.2023.1268307 (PMC10600478; doi:10.3389/fmed.2023.1268307)
Supplement: Supplementary file 1 [file Data_Sheet_1.pdf]

Supplementary Table S1. Genetic results of 8 ADPKD probands

Pedigree	Gene	Chromosome	Gene subregion	Nucleotide	Amino acid	ACMG class	ACMG evidence
A	*PKD1*	chr16:2153611	EX23 /CDS23	c.8447T>C	p.Leu2816Pro	L. Path[26-28]	PS4_Supporting+PM1+PM2+PP4
	PKD1	chr16:2160921	EX15 /CDS15	c.4247T>G	p.Phe1416Cys	Uncertain	PM1+PM2
B	*PKD1*	chr16:2161867-2161870	EX15/CDS15	c.3298_3301delGAGT	p.Glu1100Thrfs*3	L. Path	PVS1+PM2
C	*PKD1*	chr16:2160370	EX15/CDS15	c.4798A>G	p.Thr1600Ala	Uncertain	PM1
	PKD1	chr16:2158762	EX15/CDS15	c.6406C>T	p.Gln2136*	Path[29]	PVS1+PM2+PP1_Moderate+PP4
D	*PKD1*	chr16:2156228	EX19/CDS19	c.7567G>A	p.Glu2523Lys	Uncertain	PM1+PP3
E	*PKD1*	chr16:2142535	EX39/CDS39	c.11215C>T	p.Gln3739*	Path	PVS1+PM2+PP4
	PKD1	chr16:2141171	EX43/CDS43	c.11717G>C	p.Cys3906Ser	Uncertain	PM1+PP4
F	*PKD1*	chr16:2156087	IVS19/IC19	c.7703+5G>C	-	Uncertain	PM2+PP3
G	*PKD1*	chr16:2153746	EX23/CDS23	c.8312A>T	p.Glu2771Val	L. Path[30]	PM1+PM2+PM5+PP3
	PKD1	chr16:2161872	EX15/CDS15	c.3296G>A	p.Gly1099Asp	Uncertain	PM1
H	*PKD1*	chr16:2153542-2153543	EX23/CDS23	c.8515_8516insG	p.Ile2839Serfs*98	Path	PVS1+PM2+PP4
	PKD1	chr16:2159644	EX15/CDS15	c.5524C>A	p.Pro1842Thr	Uncertain	PP4+BS2

Abbreviations: L.Path, PS4, PM1, PM2; PP3, PP4, PP5, PP6, PVS1, BS2. (see appendix)

Appendix:

1. ACMG Guide Evidence Item Description and Pathogenicity

Calculation Method

Description of evidence items in ACMG/AMP guidelines (items with * are evidence items related to mitochondrial test):

PVS1: When the pathogenic mechanism of disease is loss of function (LOF), the mutation is detected as a nonfunctional mutation (nonsense mutation, frameshift mutation, classical ± 1 or 2 splicing mutations, start codon mutation, single or multiple exon deletions).

***PVS1**: large mitochondrial mtDNA deletion, at least one gene is completely deleted. For evaluation of small deletions, nonsense, and frameshift variants in protein-coding genes, refer to the updated version of PVS1.

PVS1_ Strong: PVS1 degradation.

PVS1_ Moderate: PVS1 degradation.

PVS1_ Supporting: PVS1 degradation.

PS1: It has the same amino acid change as the previously determined pathogenic variation.

PS2: Patient's new mutation without a family history (verified by parents). PS2_ VeryStrong: PS2 upgrade.

PS2_ Moderate: PS2 degradation.

PS2_ Supporting: PS2 degradation.

PS3: In vivo and in vitro function tests have identified mutations that can lead to impaired gene function

*** PS3_ Supporting**: Reliable experiments in vitro or in vivo show that the mutation destroys the function of mitochondria.

PS4: The frequency of mutation in the diseased population was significantly higher than that in the control population.

PS4_ Moderate: PS4 degradation.

PS4_ Supporting: PS4 degradation.

PM1: It is located in the hot spot mutation region, and/or in the key functional domain of known benign mutations.

PM2: Variation not found in normal control population in ESP database, 1000 person database, and EXAC database (or extremely low-frequency locus in recessive genetic disease)

* **PM2_ Supporting**: The frequency of records in the MtDB database and MITOMAP database is less than 0.00002 (0.002%, 1/50000).

PM3: In recessive genetic diseases, pathogenic variation is detected at the transposition.

PM3_ VeryStrong: PM3 upgrade.

PM3_ Strong: PM3 upgrade.

PM3_ Supporting: PM3 degradation.

PM4: protein length change caused by insertion/deletion or termination codon loss in non-repetitive region frame.

PM5: The new missense mutation leads to the change of amino acid, which has not been reported before, but the mutation of another amino acid at the same site has been confirmed to be pathogenic

* **PM5_ Supporting**: PM5 degradation.

PM6: new mutations not verified by parent samples.

PM6_ VeryStrong: PM6 upgrade.

PM6_ Strong: PM6 upgrade.

PM6_ Supporting: PM6 degradation.

PP1: Mutation and disease are separated in the same family (the mutation is detected in multiple patients in the family).

PP1_ Strong: PP1 upgrade.

PP1_ Moderate: PP1 upgrade.

PP2: For a gene, if the missense variation of this gene is the cause of a disease, and the proportion of benign variation in this gene is very small, new missense variation is found in this gene.

PP3: A variety of statistical methods predict that the mutation will cause harmful effects on genes or gene products, including conservative prediction, evolutionary prediction, splicing site impact, etc.

PP4: The phenotype or family history of the mutation carrier is highly consistent with a single-gene genetic disease.

***PP4**: The activity of ETC # enzyme in muscle, liver, and/or fibroblast is reduced (<20%) in the laboratory approved by CLIA * (or equivalent certification).

BA1: Variation with median gene frequency>5% in ESP database, 1000 person database, and EXAC database (if there is no specific gene or frequency threshold correction of variation)

* **BA1**: The top-level haploid group defines the frequency allele frequency>0.01 (1%) of variants belonging to members of the same top-level haploid group or in MtDB and MITOMAP.

BS1: The allele frequency is greater than the incidence rate of the disease

* **BS1**: The frequency allele frequency of MtDB and MITOMAP is 0.005-0.0099 (0.5% - 0.99%).

BS2: For early fully penetrance diseases, the mutation is found in healthy adults (homozygous for recessive genetic diseases, heterozygous for dominant genetic diseases, or X-linked hemizygotes)

* **BS2**: In healthy adults, especially in healthy maternal family members, the heterogeneity of variation is higher than that in the same organization of affected individuals

* **BS2_ Supporting**: BS2 degradation.

BS3: In vivo and in vitro experiments, it is confirmed that the variation has no effect on protein function and splicing

* **BS3_ Supporting**: There is no functional effect in hybrid studies or single fiber analysis.

BS4: Lack of co segregation in a family member

* **BS4**: Lack of co separation or co separation in paternal members.

BP1: It is known that the cause of a disease is a missense

mutation found in a gene due to a truncated mutation.

BP2: A known pathogenic variation of the same gene on another chromosome is found in a dominant genetic disease, or a known pathogenic variation of the same gene on the same chromosome is found in a genetic disease of any genetic model.

***BP2**: Other mutations of mtDNA previously confirmed as pathogenic are found in individuals.

BP3: Deletion/insertion in the repetitive region with unknown function did not lead to the change of gene coding frame.

BP4: A variety of statistical methods predict that the mutation will have no impact on the gene or gene product, including conservative prediction, evolutionary prediction, splicing site impact, etc.

BP5: A mutation found in a case with another molecular cause of disease

* **BP5**: Mitochondrial DNA variation found in diseases related to nuclear DNA.

BP7: synonymous variation and prediction do not affect splicing.

2. The reference database and prediction software versions are as follows:

ClinVar (2020-03-16), ESP6500 (V2), Thousand Genome (Phase3), GnomAD (v2.1.1), ExAC (r0.3.1), BPGD* (V3.1), SecondaryFinding_Var* (V1.1_2020.3), dbscSNV (1.1), SpliceAI (1.3), dbNSFP (2.9.1), SIFT, MutationTaster, PolyPhen-2, PhyloP, GERP, etc.* BPGD (BGI Phoenix genetic database)
is acomprehensive genetic disease database developed by BGI.
It is based ongene-
disease information from the OMIM database and integrates content
from various databases such as OMIM, GeneReviews, Orphanet, Genetic Home Reference, and Uniprot. The database mainly covers gene and disease names, genetic modes, clinical characteristics, and other relevant information. SecondaryFinding_Var (V1.1_2020.3) is BGI's internal variation database, containing 2,839 pathogenic and suspected pathogenic variations in 59

genes.
